# Correction: Seeding the future of nanomaterials: a comprehensive review of nanosheet-mediated growth for energy harvesting, energy conversion, and photodetection applications

**DOI:** 10.1039/d6ra90001a

**Published:** 2026-01-12

**Authors:** Attia Shaheen, Nadeem Raza, Irfan Ijaz, Aysha Bukhari, Mavra Farrukh, Mostafa E. Salem

**Affiliations:** a Henan Key Laboratory of High-Temperature Functional Materials, School of Materials Science and Engineering, Zhengzhou University Zhengzhou 450001 China; b Department of Chemistry, College of Science, Imam Mohammad Ibn Saud Islamic University (IMSIU) Riyadh Kingdom of Saudi Arabia; c School of Chemistry, Faculty of Basic Sciences and Mathematics, Minhaj University Lahore Lahore 54700 Pakistan iffichemixt266@gmail.com; d Institute of Micro-Nanoscale Optoelectronics, Shenzhen University Shenzhen 518060 China

## Abstract

Correction for “Seeding the future of nanomaterials: a comprehensive review of nanosheet-mediated growth for energy harvesting, energy conversion, and photodetection applications” by Attia Shaheen *et al.*, *RSC Adv.*, 2025, **15**, 20469–20494, https://doi.org/10.1039/D5RA01655J.

The authors regret that affiliation *a* was incorrect in the original article. The correct affiliation is as shown here, whereby Attia Shaheen is associated with Henan Key Laboratory of High-Temperature Functional Materials, School of Materials Science and Engineering, Zhengzhou University, Zhengzhou 450001, China.

The Royal Society of Chemistry apologises for these errors and any consequent inconvenience to authors and readers.

